# The Role of Aerobic and Anaerobic Training Programs on CD^34+^ Stem Cells and Chosen Physiological Variables

**DOI:** 10.2478/v10078-012-0080-y

**Published:** 2012-12-30

**Authors:** Mohammed Nader Shalaby, Mohammed Saad, Samy Akar, Mubarak Abdelreda Ali Reda, Ahmed Shalgham

**Affiliations:** 1 Department of Pathobiology key lab of Ministry of Education, Norman Bethune College of Medicine, Jilin University, China and Department of Sports Science, Faculty of Physical Education, Suez Canal University, Egypt.; 2 Faculty of Physical Education, Suez Canal University, Egypt.; 3 Department of Physical Education, College of Basic Education, Public Authority for Applied Education and Training, Kuwait.

**Keywords:** Aerobic and anaerobic training programs, CD34+ stem cells, physiological variables

## Abstract

Exercise is one of the most powerful non-pharmacological strategies, which can affect nearly all cells and organs in the body. Changes in the behavior of adult stem cells have been shown to occur in response to exercise. Exercise may act on regenerative potential of tissues by altering the ability to generate new stem cells and differentiated cells that are able to carry out tissue specific functions. The purpose of this study was to reveal the role of aerobic and anaerobic training programs on CD34+ Stem Cells and chosen physiological variables. Twenty healthy male athletes aged 18–24 years were recruited for this study. Healthy low active males and BMI matched participants (n=10) aged 20–22 years were recruited as controls. Aerobic and anaerobic training programs for 12 weeks were conducted. VO2max pulse observation was carried out using the Astrand Rhyming protocol. RBCs, WBCs, HB and hematocrit were estimated using a coulter counter, lactate by the Accusport apparatus, CD34+ stem cells by flow cytometry. VO2max was increased significantly in case of the aerobic training program compared to anaerobic one (62±2.2 ml/kg/min vs. 54±2.1 ml/kg/min). Haemotological values increased significantly in the anaerobic program when compared to the aerobic one, RBCs (5.3±0.3 and 4.9±0.2 mln/ul), WBCs (6.6±0.5 and 6.1±0.4 thous/ul), HB (15.4±0.4 and 14.2±0.5 g/de), Hematocrit (4.6±1.2 and 4.4±1.1 %), CD34+ stem cells count increased significantly in case of the anaerobic program compared to the aerobic (251.6±21.64 and 130±14.61) and sedentary one (172±24.10). These findings suggest that anaerobic training programs provoke better adaptation to exercise and stem cell counts may differ between trained and sedentary subjects. Circulating immature cells are likely to be involved in angiogenesis and repair process, both mechanisms being associated with strenuous exercise. Knowledge of the physiological effects of training on stem cells might be of potential clinical use.

## Introduction

Exercise is one of the most powerful non-pharmacological strategies, which is able to affect nearly all cells and organs in the body. In this context, a new research avenue focusing on the action of exercise on adult stem cells has emerged during the last decade. Changes in the behavior of adult stem cells from different regions, including skeletal muscle and the cardiovascular system have been shown to occur in response to exercise.

In general, exercise understood as both acute and systematic training, has been found to stimulate increases in circulating EPCS in healthy subjects and patients with cardiovascular disease, although there are few studies that lend insight into these mechanisms and signal their relationship with exercise (Witkowski, 2008)

Through its action on adult stem cells, exercise may act on the regenerative potential of tissues by altering the ability to generate new stem cells and differentiated cells that are able to carry out tissue specific functions (Kado and Thornell, 2000). Strength and power are important aspects of fitness, sport and everyday activity. However, much debate remains as to how these qualities should be evaluated. Much of the debate originates from the definition of strength and power as well as the different terminology used across laboratories. Sale (1991) defined strength as the force exerted under a given set of conditions during a maximal voluntary contraction (MVC).

He continued to define power as the rate at which mechanical work is performed under a specified set of conditions, or the product of force and velocity. Both definitions imply that strength and power are defined by conditions such as velocity, contraction type, posture and movement pattern specificity. That is strength for one task may not imply for another one. Strength and power are quite often measured in contexts dissimilar to the environment in which functional strength and power are needed (Fatourous et al., 2000).

Guyton and Hall (2006) reported the effect of athletic training on muscles. They stated that muscles that function under no load, even if they are exercised for hours, increase little in strength. On the other hand, muscles that contract at more than 50% maximal force will develop strength rapidly even if the contractions are performed only a few times each day. They also added that during muscle contraction blood flow increases about 13 fold but also the flow decreases during each muscle contraction. This decrease in flow is due to the compression of intramuscular blood vessels, but the blood flow to muscles increases during contraction.

Hawke (2005) stated that although endurance training is associated with high repetitions in low resistant exercise, significant muscle damage can occur if the duration or mode of exercise is extreme, for example, both marathon running and downhill running can lead to significant muscle fiber damage. In contrast to endurance training, resistant exercise is associated with high intensity, low repetition, high load exercise increases muscular strength, power and anaerobic capacity, with little change in aerobic power. The workloads placed on skeletal muscle during resistance training are at or near maximal capacity, and as such produce significant perturbations in skeletal muscle fibers and the associated extracellular matrix.

In a recent study, Burd et al. (2010) investigated the impact of two distinctly different exercise volumes on anabolic signaling myogenic gene expression, and rates of muscle protein synthesis (Mix, Myo, Sarg), specifically, they utilized a unilateral model in which subjects performed exercise at 90% 1RM until failure (90 FAIL), 30% 1RM in which the amount of external work was matched to 90 FAIL (30 WM), or 30% 1RM to failure (30 FAIL). They reached the conclusion that low-load high volume resistance exercise is more effective in inducing acute muscle anabolism than high load low volume or work matched resistance exercise modes.

As for training induced adaptations, exercise induced neutrophilia was shown to become progressively blunted with training (Suzuki et al., 1999), but no other study tested whether circulating HPC counts may differ between trained and sedentary subjects. Circulating immature cells are likely to be involved in angiogenesis (Reyes et al., 2002) and repair processes (Springer et al., 2001), both mechanisms being possibly associated with strenuous exercise and progressive training. Given the large use of exercise based on rehabilitation programs in several diseases, knowledge of the physiological effects of training on HPCs might be of potential clinical use.

Identification of EPCs on the cell surface expressions of various protein markers.

There is no straight forward definition of an EPC marker because these cells seem to be a heterogeneous group associated with different cell surface antigen expression profiles. The most commonly described molecules that serve as biomarkers for recognition of an EPC population include CD34+, CD133, and VEGFR2. The pioneer study of Asahara et al. (1999) recognized EPCs as CD34+ mononuclear cells (MNCs). Hematopoeietic stem cells that serve as a source of EPCs express CD34+, however this marker is also present on the surface of mature endothelial cells (Fina et al., 1990).

Human CD133 antigen is a membrane glycoprotein of which expression is related to hematopoeitic stem cell differentiation into EPCs (Urbich and Dimmeler, 2004). The third marker proposed for EPC identification is VEGFR2, a protein predominantly expressed on the endothelial cell surface. Urbich and Dimmeler (2004) and Birn et al. (2005) claimed that EPCs were positive for CD34+, CD133 and VEGFR2 markers.

CD34+ cells are multipotent progenitors that can engraft in several tissues (Krause et al., 2001), circulating CD34+ cells can be used to indirectly estimate hematopoiesis based on CD38, human leukocyte antigen (HLA) Dr, and CD33 markers.

Patrick and Stephane (2003) found CD34+ stem cell from elite triathletes to be significantly lower than in healthy sedentary subjects. They stated that the low CD34+ counts and neutopenia as well as low lymphocyte counts could contribute to the increased upper respiratory tract infections observed in these athletes. They hypothesized three explanations (1) aerobic training could induce deleterious effect on BM by inhibition of central CD34+ SC growth; (2) intense training could depress the mobilization of CD34+ SC; (3) due to aerology of the damage / repair process. They concluded that CD34+ SC quantification in elite athletes should be helpful for both basic science research and sport clinicians.

The aim of this study was to reveal the role of aerobic and anaerobic training programs on CD34+ stem cells and chosen physiological variables.

## Material and Methods

### Participants

Twenty healthy male athletes aged 18–24 years with a training history of 4–9 years were recruited for this study. Athletes had to engage in regular exercise at least 3 days/week. Healthy low active male and BMI matched participants (n=10) aged 20–22 years were recruited as controls. Control subjects could not have a recent history of regular exercise. Participants were screened and asked to fill out a health and physical activity history questionnaire.

All participants were nonsmokers, non-diabetic and free of cardiovascular, lung and liver diseases. Participants did not take any medications that affect the EPCs number or function. These include statins, angiotensin 11 receptor antagonists, ACE inhibitors, peroxisome proliferators activated receptor (PPARα) agonists and EPO.

### Testing procedures

Written informed consent was obtained from all participants and the study was approved by the University of Suez Canal Institutional Review Board. All participants engaged in a preliminary screening visit to evaluate resting blood pressure and fasting blood chemistry profile, to rule out the presence of cardiovascular disease and to obtain samples of blood for analyses and BMI testing.

All subjects were given a weight data log and instructed to weight themselves in the morning and evening and record their body mass in the log. All participants refrained from caffeine and vitamins 48 hours prior to the test. Participants were instructed to record their intake of foods for the three days before the test on a provided log.

Athletes were divided into two groups. One of the groups was subjected to an aerobic training program while the other was subjected to an anaerobic exercise program. The training program lasted for 12 weeks. For each group, a protocol consisted of a warm up, main part and a cool down. Maximal oxygen uptake (VO2max) is the maximal rate at which the body can consume oxygen during exercise (Davis et al., 1976). The test of maximal oxygen uptake is an example of both low and high intensity exercise (50 watt increment, 3 min stage protocol in aerobic exercise 25 watt each as for anaerobic exercise 100 watt increment, 30 second stage protocol by adding 50 watt each). The incremental exercise is applied by a bicycle ergometer against increasing loads until volitional fatigue. The Astrand Rhyming performed on a nomogram for estimating VO2max to use the nomogram for cycle ergometry exercise; a line is drawn connecting the gender specific heart rate to the specific workload (kg/min). When this straight line intersects the diagonal VO2max line represents the VO2max value.

The predicted VO2max value is obtained by connecting the point on the VO2 scale with the corresponding point, on the pulse rate scale. Where the line intersects the VO2max scale is the estimate of the individual’s VO2max.

As for lactate estimation, the Accusport apparatus was used. We usually determine lactate in whole blood rather than plasma or serum. Accusport is a portable device that measures lactate within a minute of applying a drop of blood from a fingertip or earlobe. When measuring lactate in the blood, it is necessary to remember that it takes 4–5 minutes to peak.

VO2max value was obtained using the Astrand Rhyming nomogram. Rbcs, Wbcs, Hb and hematocrit value were estimated using the coulter counter method.

Blood samples (5ml) were drawn, with the subject in the sitting position, at rest, from the anticubital vein into sterile tubes containing EDTA, for blood cell counts using the coulter counter (Beckman).

The human erythrocyte is the mature unit of the red blood corpuscle. It is a circular, elastic non-nucleated, biconcave disc, whose primary function is the transport of hemoglobin. Hemoglobin is a protein of 200 to 300 million nearly spherical molecules in each red blood cell, having a molecular weight of 64.458 based on the chemical structures of its alpha and beta chains. Hematocrit (the packed cell volume) is the percentage of the total volume of whole blood that is occupied by packed red blood cell when a known volume of whole blood is centrifuged at a constant speed for a constant period of time. White blood corpuscle (leukocyte) includes all white cells of the blood, lymphocytes, monocytes, neutrophil, basophil and eosinophil (Guyton and Hall, 2006).

### Circulating progenitor cell number

CD34+ (HPc, hematopoietic progenitor cell number) was determined by flow cytometry. For this assay 0.5 ml of blood was collected into an EDTA-coated tube. Mononuclear cells were separated via density centrifugation. Cells were washed and counted with a hemocytometer. Mononuclear cells were immunostained with monoclonal anti-bodies against human CD34+ for each group of analyses, one set of control tubes for machine calibration was generated. Flow cytometry was performed in a laboratory. The forward side scatter plot was used to identify lymphocyte gate. 100.000 events per sample were acquired. Total cell count was averaged. The principle, clinical applications precautions and methodology are as follows:

### IOTest CD34+ PE

The use of this fluorochrome-conjugated antibody permits the identification and numeration of cell populations expressing the CD34+ antigen present in human biological samples using flow cytometry.

### Principle

This test is based on the ability of specific monoclonal antibodies to bind to the antigenic determinants expressed by leucocytes. Specific staining of the leucocytes is performed by incubating the sample with the IOTest reagent. The red cells are then removed by lysis and the leucocytes, which are unaffected by this process, and then, analyzed by flow cytometry. The flow cytometer measures light diffusion and the fluorescence of cells. It makes possible the delimitation of the population of interest (cells) within the electronic window defined on a histogram, which correlates the orthogonal diffusion of light (Side Scatter or SS) and the diffusion of narrow angle light (Forward Scatter or FS). Other histograms combining two of the different parameters available on the cytometer can be used as supports in the gating stage depending on the application chosen by the user. The fluorescence of the delimited cells is analyzed in order to distinguish the positively stained events from the unstained ones. The results are expressed as a percentage of positive events in relation to all the events acquired by the gating.

### Procedure

Note: The procedure below is valid for standard applications. Sample and/or Versa Lyse volumes for certain Beckman Coulter applications may be different for each sample analyzed. In addition to the test tube, one control tube is required in which the cells are mixed in the presence of the otypic control (Ref. A07796).

1. Add 20 μL of specific IOTest conjugated antibody to each test tube, and 20 μL of the isotypic control to each control tube.
2. Add 100 μL of the test sample to both tubes. Vortex the tubes gently.
3. Incubate for 15 to 20 minutes at room temperature (18 – 25°C), protected from light.
4. Then perform lysis of the red cells, if necessary, by following the recommendations of the lysis reagent used. As an example, if you wish to use VersaLyse (Ref. A09777), refer to the leaflet and follow preferably the procedure called “with concomitant fixation”, which consists of adding 1 ml of the “Fix-and-Lyse” mixture prepared extemporaneously. Vortex immediately for one second and incubate for 10 minutes at room temperature, protected from light. If the sample does not contain red cells, add 2 ml of PBS.
5. Centrifuge for 5 minutes at 150 × g at room temperature.
6. Remove the supernatant by aspiration.
7. Resuspend the cell pellet using 3 ml of PBS.
8. Repeat step 5.
9. Remove the supernatant by aspiration and resuspend the cell pellet using:
  − 0.5 ml or 1 Ml of PBS plus 0.1% of formaldehyde if the preparations are to be kept for more than 2 hours and less than 24 hours. (A 0.1% formaldehyde PBS can be obtained by diluting 12.5 μL of the IOTest 3 Fixative Solution (Ref. A07800) at its 10X concentration in 1 Ml of PBS).
  − 0.5 Ml or 1 Ml of PBS without formaldehyde, if the preparations are to be analyzed within 2 hours.

In all cases, keep the preparations between 2 and 8°C and protected from light.

Body height and body mass were recorded and body mass index. BMI (kg/m2) was calculated for all subjects. The BMI for the athletes equaled 22±1.4 and for control subjects 23±2.2.

### Statistical Analysis

Student’s t tests were used to determine the differences between athletes and control groups and between aerobic and anaerobic groups when exercise data were found to not meet the assumption of normality, the non-parametric Mann Whitney U test (Wilcoxon rank sum test) was used to compare differences between groups. In these cases, for descriptive data the median (Lowest value-highest value) is displayed. Differences between groups were tested using the analysis of variance (ANOVA). For parameters with non-normal distributions non parametric Spearman correlation coefficients were used. The F test was used to test 3 groups. An α level of 0.05 was used to indicate statistical significance.

### Aerobic Training Program After Dr. Phil Esten (2010)

Early season phase: this training phase takes place during the first 4 weeks of a 12 week program. A typical week consists in following aerobic-paced mileage (3miles) on Monday and Wednesday, with a hard up-tempo workout on Tuesday (anaerobic threshold and race pace), a lighter up-tempo workout on Thursday, 20–40 minutes of slow running on Friday, a race on Saturday and Sunday is a day off, weight training is performed on Monday and Wednesday. During the early season phase the athletes should have one very long run every 14 days and the workouts should progress in duration every 2 weeks by 5 minutes. Intensity = HR of 140–150beats/m. Midseason phase should begin after 4 weeks and continue through week 8. During this phase, the runners should have one very long, aerobic paced run every 14 days (20–50%) longer than their run of the week. Every 2 weeks running time increases by 5 minutes, weight training continues on Monday and Wednesday. The difference with this phase as compared to the early phase is that both duration and intensity of the work are at a higher level.

The final phase covers the last 4 weeks of a 12 week training program. It is important to continue with aerobic pace runs to ensure stride efficiency. By maintaining training duration (5 percent cut back per week), increasing the rest interval, and slightly decreasing the intensity of the repetitions, the runner recovers from the midseason work and performs at optimal levels.

### Anaerobic Training Program After Tom Green (2003)

Training 3 to 4 times per week: on Monday, Tuesday, Thursday and Friday. Saturday is reserved for a single specific workout at high intensity.

-1sr workout: 20–45 minutes of an active warm-up, max velocity sprint, plyometric/bounding and a cool down of 10–15 minutes.
-2nd workout: 20 minutes of a warm-up, main workout and general conditioning, 10–15 minutes of a cool down.
3rd workout weights using high load (70%1RM)
-Saturday workout is typically late morning or early afternoon.

As the season progresses the workout program gets increasingly more technical, specific and fine-tuned, intensity increases reaching 170 beats/min. After a proper warm up for 20 minutes, the first workout consists of 30 minutes to an hour of work at maximum velocity sprint and /or plyometric.

The second workout throughout the week:

Monday: the 30 s sprint, then 10 minutes of a rest, afterwards sprinting at 80% up a hill for around 100 meters, then a cool down.
Tuesday: a warm up, standing in place – running, arm swings, performed with dumbbells (5–25 Lbs) then a single leg squat, with a 2 minutes rest between each set.
Wednesday: rest and recovery.
Thursday: a warm up, the workout in place, weighted arm swings 10 sets of 60 s on and 60 s off, and then cool down.
Friday: workout as on Monday then a cool down
Saturday: workout is used for active recovery

As the athlete adapts to training the rate of progression can be increased

### Subject characteristics

Twenty athletes and ten low active control males participated in the study which consisted of the 12 week aerobic and anaerobic training program. Groups were matched for age, body mass and body height (Table 1). Non-significant changes in basic characteristics of athletes and control males were observed. Heart rate and VO2 max showed significant changes (Table 1), as expected athletes had a lower heart rate compared to the control group. Physical activity questionnaire data revealed that athletes exercised an average of 5±0.5 days a week for 5±0.2 years. The control group was not engaged in regular exercise, nor did they have a recent history of physical activity

## Discussion

Tables 1 and 2 present lower values of lactate after aerobic and anaerobic training programs what means a better level of fitness. Lactate is the end product of the anaerobic carbohydrate breakdown. It is the metabolite displaying the most spectacular concentration changes in muscle and blood with exercise. As a result, its measurement offers a wealth of information regarding the effect of exercise on metabolism. Lactate is usually determined in blood plasma (Mougios, 2006). Peak lactate concentration after short maximal exercise is reached after 3–5 minutes.

Programming training based on blood lactate concentration is superior to programming on heart rate alone, because lactate relates directly to muscle metabolism and muscle adaptations. Thus, one could use lactate to determine training intensities at the beginning of a training program, monitor training through heart rate on a daily basis, and relate to lactate every few weeks to control the intensity. Most scientists agree that exercise intensities below 4mmol/L are most effective in improving aerobic endurance, cardiac function and the lipidemic profile (Mougios, 2006; Greenhaff and Timmons, 1998).

Barrett et al. (2010) stated that blood consists of a protein rich fluid known as plasma, in which cellular elements are suspended: white blood cells, red blood cells and platelets. The normal total circulating blood volume is about 8% of the body mass (5600 ml in a 70 kg man). About 55% of this volume is plasma. Red blood cells, white blood cells and platelets are formed in the bone marrow, which is actually one of the largest organs in the body, approaching the size and weight of the liver. Hematopoietic stem cells (HSCS) are bone marrow cells that are capable of producing all types of blood cells. They differentiate into committed stem cells (Progenitors cells). The Hscs are derived from uncommitted, tot potent stem cells that can be stimulated to form any cell in the body. Adults have a few of these, but they are more readily obtained from the blastocysts of the embrio.

Robergs and Roberts (1997) stated that the main functions of the cellular components of blood are the transport of oxygen and carbon dioxide, blood clotting, acid base buffering immune functions and tissue repair and destruction. Therefore, functions of plasma (liquid components) are as follows: blood clotting, circulating of cellular components and their contents, heat transfer and thermoregulation, water exchange and transport, circulation of hormones, acid base buffering, circulation of metabolites, nutrients and waste products.

Burge et al. (1993) and Gillen et al. (1991) reported that acute effect of exercise on blood is to cause a release of fluid from the vascular component, which decreases the volume of plasma and blood. This fluid loss from plasma decreases plasma volume and causes hematocrit and plasma metabolite concentration to increase, which is termed hemoconcentration. In fact, a significant hemoconcentration occurs when a person moves from a supine to a vertical position. The added hemoconcentration of exercise is predominantly confined to the transition from rest to exercise. This response is followed by a more gradual hemoconcentration that occurs with increases in exercise intensity. And these changes are greater during higher blood pressure associated with resistance exercise than during more prolonged dynamic exercise.

Spriet et al. (1986) added that prolonged exercise involving sweating increases fluid loss from the body, and the degree of hemoconcentration can be evaluated by either directly measuring plasma volume or estimating relative changes in plasma volume from hemoglobin and hematocrit measurements. Also blood viscosity increases above what would be expected for hemoconcentration effects. In addition, there is destruction of erythrocyte, termed hemolysis, which increases plasma hemoglobin concentration (Zierler et al., 1992). This was in accordance with the increased cellular changes after training programs due to hemoconcentration (Table 3).

Endurance training increases the volume of blood. The ventricle can hold and contributes to its maximum stroke; ventricular thickness is usually slightly increased. The blood cells, Rbs, Wbcs, platelets and Hematocrit as well as hemoglobin are slightly increased together with stem cells SC, CD34+ (Tables 2, 3).

As for the adaptive response to anaerobic exercise, blood cellular components of Rbcs, Wbcs, Hct and haemogbin numbers and contents increased together with increase CD^34+^ SC compared to aerobic one and control CD^34+^ (25,6±21,64) (130±14,61) and 170±21.10 (Tables 2 and 3), this was in accordance with the results of Bonsignore et al. (2002; 2010) and, Mobius–Winkler et al. (2009).

Robergs and Roberts (1997) stated that the most important chronic adaptation increasing long term muscular endurance is an increase in number and size of mitochondria. An increased mitochondrial volume would also provide skeletal muscle with the ability to increase VO_2max_. However, cardiovascular adaptations are also involved in increasing VO_2max_ after training. Table 4 presents increased VO_2max_ after the aerobic training program compared to control and anaerobic one.

Amani and Mohamed (2011) reported the effect of endurance and resistance training on CD^34+^ / CD^45+^ Stem cells, VO_2max_, certain physical variables and time of the 1500 m run. They came to the results that there was a significant increase in post measures in accounting of CD^34+^ / CD^45+^ Stem cells, VO_2max_ and time of 1500 m run with regard to endurance and resistance training. They concluded that a two month training program can improve values of physical variables and time together with increased stem cells among young runners

Resistance exercise stimulates the synthesis of skeletal muscle proteins (West et al., 2009), which is expressed as muscle hypertrophy. It has recently been established that myofibrillar (My) protein synthesis is already maximally stimulated at 60% 1RM, in the post absorptive state, with no further increase at higher load intensities (75 – 90 % 1RM) (Kumar et al., 2008).

Additionally, performance of low load contraction (20 1MR) with vascular occlusion is sufficient to induce an increase in mixed muscle (Mix) protein synthesis (Fujita et al., 2007).

Burd et al. (2010) reported that low-load high volume resistance exercise is more effective in inducing acute muscle anabolism than high-load low volume or work matched resistance exercise modes. Fifteen young men (21±1 years), performed 4 sets of unilateral leg extension exercise at different loads and/or volumes. 90% of (1RM) until volitional failure (90 FAIL) 30% 1RM work matched to 90% fail (30 wM), or 30% 1RM performed until volitional failure (30 FAIL).

Regular physical activity is associated with enhanced endothelial function which has been related to lower incidence of cardiovascular disease (Delp et al., 1993; Delp 1995; Hambrecht et al., 2003; Haram et al., 2006).

Bonsignore et al. (2002) suggested that increased HPCS reflect adaptation to recurrent, exercise-associated release of neutrophils, stress and inflammatory mediators, indicating modulation of bone marrow activity to habitual running. Laufs et al. (2004) measured EPCS in mice and patients with stable CAD. Mice engaged in 3 weeks of voluntary wheel running and humans underwent a 4 week training program of bicycle ergometer endurance exercise (60– 80% of VO_2max_), strength exercise, and walking. EPC number was significantly increased in the blood, bone marrow of mice after 7 days of exercise which persisted for the 28 days of the training program. In human the number increased 78 ± 34% compared to the initial level. EPC apoptosis was found to decrease 41 ± 11 % after training. As for Steiner et al. (2005) who conducted a 12 week exercise program in patients with asymptomatic coronary artery disease (CAD), results showed a 2.9 ± 0.4 fold increase in circulating EPCs in the exercise group. This increase was correlated with an increase in flow mediated dilation and no synthesis.

In another study, Sandri et al. (2005) analyzed the responses of circulating CD^34±^/KDR^±^, their number and function in three groups of patients suffering from ischemia. The training program lasted for 4 weeks. Increases in CPC in all three groups were accompanied by an increase in CXCR4, and VEGF. They concluded that the ischemic exercise groups appeared to increase VEGF, which may have stimulated the increase in CPC numbers. Thijssen et al. (2006) reported no change in CD^34+^/ KD^±^ cells in younger and older healthy participants following 8 weeks of cycle exercise for 20 minutes 3 time per weeks at 65% of heart rate reserve. Therefore, exercise may not increase EPC number in healthy individuals. Hoetzer et al. (2007) and Vasankari et al. (1998) reported that exercise may improve the number and function of EPCS while improving oxidative stress status.

## Conclusion

### It may be concluded that

- Knowledge of the physiological effects of training on stem cells might be of potential clinical use.
- Cardiovascular adaptations are involved in increasing VO_2max_ after training.
- Lactate concentration was decreased in case of aerobic and anaerobic training compared to the control one meaning a better level of fitness, because lactate relates directly to the muscle metabolism and muscle adaptation.
- HB, RBCs, WBCs and hematocrit value were increased after anaerobic training compared to aerobic one due to stress.
- CD^34+^ SC counts were increased in peripheral blood of anaerobic training when compared to the aerobic one and control due to stress; it indicatd better adaptation to exercise and modulation of bone marrow activity to anaerobic training.

## Figures and Tables

**Table 1:** Basic characteristics

Variable	Athletes N=20	Control N=10
Age (yr)	21.6 ± 1.83	20.6 ± 0.89
Body Height (cm)	179 ± 2.78	178.8 ± 1.92
Body Mass (kg)	75 ± 3.16	74 ± 1.5
BMI	22 ± 1.4	23 ± 2.2
Heart rate (count/m)	68 ± 2.3	74 ± 2.1
VO_2max_(ml/kg)	52 ± 1.8	36 ± 1.7
Lactate (mmol/L)	1.1 ± 0.02	1.2 ± 0.03

Values are means ±SD p<0.05, BMI = body mass index

**Table 2:** Haematopoietic stem cells for control, aerobic exercise training and anaerobic training courses of exercise for 12 weeks in the resting stages and Lactate

Variable	Control	Aerobic training	Anaerobic training	*p*
CD^34+^S cells	172.0 ± 24.10	0.015 ± 14.61	251.6 ± 21,64	0.003
Lactate (mmol/L)	1.2 ± 0.3	0.002 ± 0.1	0.9 ± 0.2	0.012

**Table 3:** Haematological values of Rbcs, Wbcs, HB and hematocrit (PCV) after aerobic and anaerobic training program (at rest) and control

Variable	Control	Aerobic training	Anaerobic training	*p*
RBCs (million/ul)	4.7 ± 0.9	4.9 ± 0.2	5.3 ± 0.3	0.015
WBCs (thousands/ ul)	4.8 ± 0.7	6.1 ± 0.4	6.6 ± 0.5	0.002
HB (g/dL)	12.8 ± 0.8	14.2 ± 0.5	15.4 ± 0.4	0.004
Hematocrit (%)	42 ± 3.2	44 ± 1.1	46 ± 1.2	0.011

**Table 4:** The variation in VO_2max_ of participants : healthy sedentary and after aerobic and anaerobic training programs

Participants	VO_2max_ (mL/kg/min)
Healthy sedentary (mL/kg/m)	36 ± 1.7
Aerobic training program (mL/kg/m)	62 ± 2.2
Anaerobic training program	54 ± 2.1

The results are expressed as mean ± SD (p<0.05)
